# Macrophage and Tumor Cell Cross-Talk Is Fundamental for Lung Tumor Progression: We Need to Talk

**DOI:** 10.3389/fonc.2020.00324

**Published:** 2020-03-11

**Authors:** Poonam Sarode, Martina Barbara Schaefer, Friedrich Grimminger, Werner Seeger, Rajkumar Savai

**Affiliations:** ^1^Max Planck Institute for Heart and Lung Research, Member of the German Center for Lung Research (DZL), Member of the Cardio-Pulmonary Institute (CPI), Bad Nauheim, Germany; ^2^Department of Internal Medicine, Member of the German Center for Lung Research (DZL), Member of the Cardio-Pulmonary Institute (CPI), Justus Liebig University, Giessen, Germany; ^3^Frankfurt Cancer Institute (FCI), Goethe University, Frankfurt am Main, Germany

**Keywords:** lung cancer, tumor microenvironment, tumor-associated macrophages, cytokines, chemokines

## Abstract

Regardless of the promising results of certain immune checkpoint blockers, current immunotherapeutics have met a bottleneck concerning response rate, toxicity, and resistance in lung cancer patients. Accumulating evidence forecasts that the crosstalk between tumor and immune cells takes center stage in cancer development by modulating tumor malignancy, immune cell infiltration, and immune evasion in the tumor microenvironment (TME). Cytokines and chemokines secreted by this crosstalk play a major role in cancer development, progression, and therapeutic management. An increased infiltration of Tumor-associated macrophages (TAMs) was observed in most of the human cancers, including lung cancer. In this review, we emphasize the role of cytokines and chemokines in TAM-tumor cell crosstalk in the lung TME. Given the role of cytokines and chemokines in immunomodulation, we propose that TAM-derived cytokines and chemokines govern the cancer-promoting immune responses in the TME and offer a new immunotherapeutic option for lung cancer treatment.

## Introduction

Worldwide, Lung cancer is responsible for the highest number of cancer-related death in men and women (1). The 5-year survival rate in metastatic lung cancer (5%) is much lower than primary lung cancer (56%), colon (64.5%), breast (89.6%), and prostate (98.2%) cancer. Only 16% of lung cancer is diagnosed in early stages (2).

The first line of treatment in lung cancer is surgery, but most clinically detected cases are inoperable, and the chances of missing micro-metastasis and recurrence are high. When surgical intervention is not possible, then chemotherapy and radiotherapy are the next potential options, these therapies exert a devastating effect on normal tissue homeostasis and reduce health-related quality of life. An upcoming molecular targeted therapy targeting epidermal growth factor receptor (EGFR) and anaplastic lymphoma kinase (ALK), improved treatment regimen in patients with these detectable mutations. Still, for a large group of lung cancers, molecular alterations have not been shown as effective for molecularly targeted therapies. Traditionally, immunotherapy showed marginal success in lung cancer. Recently, immune checkpoint blockers targeting cytotoxic T-lymphocyte-associated protein 4 (CTLA4) and anti-programmed cell death protein 1 (PD-1) have shown results in lung cancer treatment, but only a subset of the patients achieved a strong response with minimum toxicity in these immunotherapies (3–6), which is attributed to the fact that the lung tumor cells acquire large numbers of somatic mutations, and therefore induce tumor immune evasion by suppressing immune cell-mediated immunosurveillance via multiple mechanisms, such as secretion of pro-tumor cytokines, dysfunctional antigen expression, and inactivation of T cell activation (7–10). Thus, to develop new targeted therapies, future studies should be oriented toward the analysis of the tumor-infiltrating immune cells' landscape in the tumor microenvironment (TME) and how this contributes to lung carcinogenesis.

The driver mutations in tumors operate together and direct changes in the TME, especially in tumor-infiltrating immune cells (11). The driver mutations in lung cancer are gene mutations in EGFR and KRAS proto-oncogene (KRAS), ALK rearrangements, and altered MET proto-oncogene (MET) signaling (12). Notably, the extensive immunogenomic analysis of more than 10,000 samples from The Cancer Genome Atlas comprising 33 diverse cancer types displayed a more prominent macrophage signature with T helper (Th) 1 cell suppression and an M2-like macrophage response in tumors with gene mutations in EGFR, KRAS, and KRAS G12 (13). The infiltration of macrophages was found to be increased in lung tumor area when compared to the non-tumor area. Currently, there is no consensus in the literature on whether the high density of macrophages is detrimental or beneficial in lung cancer, while few recent reports demonstrated the correlation between prognosis and patient survival with the density of particular phenotype of macrophages. The prolonged patient survival correlated with a high density of M1 macrophages, while poor prognosis correlated with a high density of M2 macrophages in tumor islets (14–19). Evidences suggest that cytokines secreted by TAMs induce hyperproliferative, anti-apoptotic, and metastatic responses in lung cancer, offering a potential immunotherapeutic option for its treatment (20–22).

## Tumor Associated Macrophages (TAMs)

Macrophages are a plastic, heterogeneous group of cells. Besides providing the innate immune response against invading pathogens, they play an essential role in maintaining tissue integrity and homeostasis. Macrophages encounter diverse microenvironmental signals, which can alter their transcriptional program and role, based on the location and distinct gene expression profiles. Functional and phenotypic dysregulation has been associated with a wide range of chronic inflammatory and autoimmune diseases, including cancer. Classically, macrophages divided into two major types, classical macrophage activation (M1) promotes a pro-inflammatory response, while alternative macrophage activation (M2) stimulates an anti-inflammatory response (23). Recently, according to specific cytokine stimulation conditions, M1 macrophages are subdivided into M (lipopolysaccharide [LPS]), M (LPS + interferon [IFN]γ), and M (IFNγ). M2 macrophages are subdivided into M (interleukin [IL] 4), M (immune complexes [Ic]), M(IL10), M (glucocorticoids [GC] + transforming growth factor β [TGFβ]), and M (GC) (24). These different types of macrophages drastically differ in their intrinsic transcription factors, metabolism, surface receptors, and secretory molecules, such as cytokines, chemokines, and growth factors, etc.

Tumor cell-macrophage crosstalk drives phenotypic and functional changes in both cell types. An intrinsic and extrinsic molecular patterning of tumor cells influences infiltration and activation of macrophages via multiple mechanisms: (i) Secretome of tumor cells shift the transcriptional program responsible for M1-like TAM activation to M2-like TAMs. Tumor cells derived Colony Stimulating Factor 1 (CSF1) and C-C motif chemokine ligand (CCL) 2 leads to increased infiltration of macrophages in TME, which later increased angiogenesis by stimulating the secretion of vascular endothelial growth factor (VEGF) (25). Tumor cells-macrophage co-culture increases expression of IL10, IL12, IL6, TNF, CCL5, CCL22, and CSF1 in macrophages, thereby inducing M2-like polarization (26). (ii) Apoptosis of tumor cells induces the activation of M2-like TAMs or suppress activation of M1-like TAMs (27). Apoptotic tumor cell-derived sphingosine-1-phosphate (S1P) and microRNA-375 alter macrophage polarization (28, 29). (iii) The alteration in macrophage function by necrotic tumor cells is still not very well elucidated. A study by Reiter et al. suggests that necrotic tumor cells promote the anti-tumor function of macrophages by increased production of nitric oxide (NO) (30). Another study by Brouckaert et al. suggests that phagocytosis of necrotic tumor cells by macrophages does not induce the production of inflammatory cytokines (31). Tumor cell-derived colony stimulating factor 1 (CSF1) promotes macrophage infiltration in the necrotic tumor area (32–34). These TAMs further support angiogenesis and invasion, and more interestingly, their high-density associated with reduced relapse-free survival (35, 36). (iv) Hypoxic tumor environments attract monocyte/macrophages followed by the differentiation and production of hypoxia-inducible factor (HIF) 1α and HIF2α, which then control the transcription of genes associated with tumor promotion processes, such as angiogenesis. Neuropilin 1 (NRP1) mediate hypoxic TME-induced activation and the pro-tumoral function of TAMs in cervical cancer (37). (v) The tumor cell-mediated metabolic shift in macrophage phenotype activates M2-like TAMs in TME. Through the mechanistic target of rapamycin kinase (mTOR) inhibition, TAMs from hypoxic areas show a decrease in glycolysis and increase in endothelial glucose availability, thereby disturbing a compact tumor vasculature to undergo invasion and metastasis (38). (vi) TAMs maintain an immunosuppressive phenotype by receiving polarization signals from tumor cells. IL1R and MYD88 mediated inhibitor of nuclear factor kappa B kinase subunit beta (IKBKB) and NFKB1 signaling cascade maintain M2-like phenotype in TAMs (39).

On the other hand, TAMs establish a pro-tumor microenvironment that influences the origin, progression, dissemination, and drug resistance of tumor cells in several ways, such as:(i) TAMs promote tumor cell growth and metastasis by secreting EGF (40), matrix metallopeptidase (MMP)s (41, 42), Wnt family member (WNT) 5A, cathepsin B, semaphorin 4D and IL1β (43). (ii) TAMs-derived migration inhibitory factor (MIF) induce DNA damage and immune escape by suppressing tumor protein P53 (TP53) activity (44). (iii) TAMs in hypoxic regions adapt to low oxygen tension by expressing HIF1α and subsequently secrete angiogenic factors, such as VEGF, IL8, cytochrome C oxidase assembly factor (COX2), and MMP9 (45). TAMs also increase tumor hypoxia and aerobic glycolysis (46). (iv) To support invasion and metastasis of tumor cells, TAMs induce epithelial-mesenchymal transition (EMT) in tumor cells via secretion of MMPs (47). (v) TAMs establish a pro-tumor anti-inflammatory environment by the recruitment of Th2 cells and regulatory T cells (48). (vi) TAMs play a part in T cell anergy and inhibition of the activation and growth of naïve T cells (49, 50). (vii) TAMs induced autocrine IL10 signaling pathway drives M2-like TAMs polarization to suppress anti-tumor response in TME (51). (viii) TAMs induce intrinsic activation of the immune checkpoint protein PDL1, which by binding to PD1 on T cells, leads to cytotoxic T cells senescence, exhaustion, and apoptosis (52).

## Cytokines and Chemokines–Diagnostic and Prognostic Biomarkers in Lung Cancer

**Figure 1:**
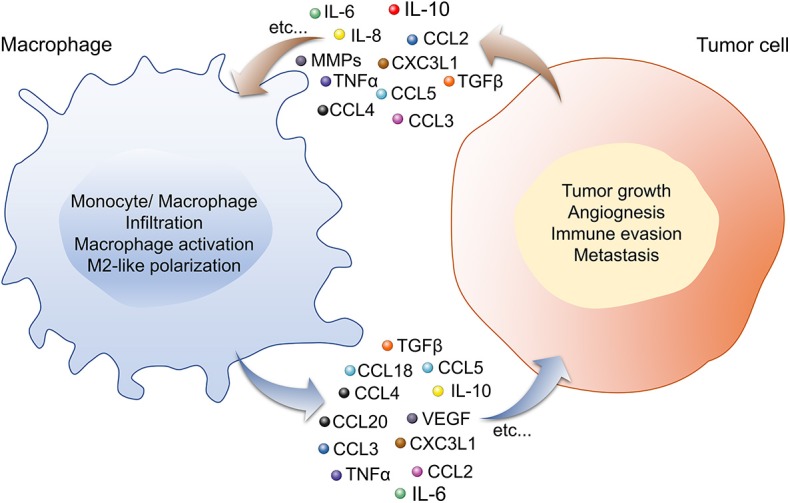
Macrophage-Tumor cells crosstalk via cytokines and chemokines through an autocrine and paracrine manner is important for lung cancer development. In the TME, cytokines, and chemokines secreted by macrophages (IL-6, -10, CCL-2, -3, -4, -5, -18, -20, CX3CL1, TGFβ, VEGF, TNFα, etc.) and tumor cells (IL-6, -8, -10, TNFα, CCL-2, -3, -4, -5, -18, -20, CX3CL1, MMPs, etc.) induce phenotypic and functional changes in both the cell types. Macrophage secretome influences tumor growth, angiogenesis, invasion, metastasis, and immune evasion by the tumor cell, while secretory factors from tumor cells regulate monocyte/macrophage infiltration, activation and polarization toward pro-tumor M2-like TAMs phenotype. Abbreviations: CCL, chemokine ligand; CXCL, chemokine (C-X-C motif) ligand; IL, interleukin; MMPs, matrix metalloproteinase; TGFβ, transforming growth factor β; TNFα, tumor necrosis factor α; VEGF, vascular endothelial growth factor.

Although tumor cell-TAM crosstalk is dependent on many factors, secreted factors (such as cytokines, chemokines, etc.) play a significant role in the crosstalk. Cytokines and chemokines are low molecular weight proteins, mainly produced by macrophages and lymphocytes. They mediate intra- and extra-cellular communication as hormones and neurotransmitters through an autocrine, paracrine, and endocrine manner. Upon binding to specific cell surface receptors, they regulate a variety of cellular processes, such as local and systemic anti- and pro-inflammation, cellular proliferation, metabolism, chemotaxis, and tissue repair, etc. In the TME, the primary role of these factors is to regulate the tumor immunity cycle. Cytokines and chemokines produced by tumor-infiltrating immune cells play a significant role in tumor development, progression, metastasis, and therapy resistance; therefore, they widely used as diagnostic and prognostic biomarkers in the treatment of cancer. As shown in Table 1, most common cytokines and chemokines used in the therapeutic management of lung cancer are IL6, tumor necrosis factor α (TNFα), IL10, IFNγ, IL2, IL22, IL32, IL37, IL8, CCL2, C-X3-C motif chemokine ligand (CX3CL1) (53–59, 64, 82–85), among which macrophages are the major source of IL6, TNFα, IL10, IL8, CCL2, and CX3CL1 [(86, 87); Figure 1]. CCL2 and CX3CL1 receive special attention in chemokine biology, because of their unique phenotypic and functional properties. The decades of extensive research in the field of cytokines and chemokines in cancer development published outstanding research and review articles. Therefore, in this review, we summarized the published literature from the year 2000 to the year 2019, specifically focusing on the role of IL6, TNFα, IL10, CCL2, CX3CL1, IL8 in the macrophages-tumor cells crosstalk; leading to lung cancer development and progression.

**Table 1:** Prognostic value of cytokines and chemokines in the therapeutic management of lung cancer and their main source of production.

**Cytokine and chemokine**	**Main source**	**Studies predicting prognostic purpose in lung cancer**	**Sample**
IL6	Macrophage Th cells Fibroblasts	11 (53–63)	Blood BALF Pleural effusion
TNFα	Macrophage	6 (14, 55, 57, 58, 64, 65)	Blood BALF
IL10	Macrophages Monocytes Th cells DCs	8 (53, 55, 58, 59, 61, 66–68)	Blood Serum, Saliva
IFNγ	Activated–T cells Activated—NK cells	3 (56, 62, 64)	Blood Serum Plasma
IL2	Activated–CD4^+^ T cells –CD8^+^ T cells	3 (69–71)	Blood Serum
IL22	Th17 cells RORγ (T)^+^ Lti cells, NCR1^+^ cells	2 (72–74)	Blood Serum Tissue
IL32	NK cells T cells Epithelial cells Blood monocytes	2 (75, 76)	Tissue
IL37	Monocytes DCs	1 (77)	Tissue
IL8	Macrophages	5 (54–56, 58, 59)	Blood BALF Serum Saliva Plasma, Sputum
CCL2	Macrophages Monocytes	3 (61, 78, 79)	Serum Tissue
CX3CL1	Macrophages Microglia Activated endothelial cells Neurons	2 (80, 81)	Tissue

*CCL2, C-C motif chemokine ligand 2; CX3CL1, C-X3-C motif chemokine ligand 1; DCs, Dendritic cells; IFNγ, Interferon gamma; IL, Interleukin; Lti, Lymphoid-tissue inducer; NK, Natural killer cells; NCR1, Natural cytotoxicity triggering receptor 1; RORγ, RAR-related orphan receptor gamma; Th, T helper cells; TNFα, Tumor necrosis factor alpha*.

## Cytokines

### IL6

IL6 plays a significant pro-inflammatory role under many physiological and pathological conditions in multiple cell types (88). Although many different cell types secrete IL6, macrophages are one of the major sources. IL-6 exerts its effects after binding to ligand-binding IL-6 receptor (IL6R) α chain (gp80, CD126) and the signal-transducing component gp130 (CD130) in an autocrine and paracrine manner. IL6 is a double-edged sword in the tumor microenvironment. Several studies demonstrated the role of IL6-mediated pro-proliferative, anti-apoptotic, angiogenic, metastatic, and immunosuppressive responses in tumor development and progression. While other studies demonstrated the role of IL6 in promoting anti-tumor immunity through the stimulation of proliferation, survival and trafficking of T cells to lymph nodes and tumor sites, where T cells effectively shift tumor-suppressive state to responsive state to inhibit tumor growth and progression (89, 90). An increased level of IL6 correlates with the poor prognosis and survival of lung cancer patients (54, 60). TAM-derived IL6 plays a role in progression, invasion, angiogenesis, EMT, immune cell infiltration, and cancer stem cell (CSC) development and maintenance, through multiple unexplored molecular mechanisms (91–93). The activation of signal transducer and activator of transcription (STAT) 3 by TAM-derived IL6 in the lung TME considered as the prime mechanism responsible for the development of mouse lung tumor model and crosstalk with small cell lung cancer (SCLC) cell lines (94, 95). Phosphatidylinositol-4,5-Bisphosphate 3-Kinase/AKT Serine/Threonine Kinase 1 (PI3K/AKT) signaling is another pathway engaged by TAM-derived IL6 to influence growth of lung cancer cell line, A549 (96). TAM-derived IL6-mediated STAT3 signaling pathway also found to increase the proliferation of human cancer stem cells (97). In different phases of lung cancer development and its therapeutic management, IL6 drives multiple molecular mechanisms responsible for the epithelial-mesenchymal transition (EMT) (98, 99) and therapy resistance, such as infiltration of pro-tumor macrophages after irradiation through the upregulation of CCL2/CCL5 *in vitro* human and *in vivo* mouse lung tumor models (100). Therefore, the blockade of IL6 reprograms the TME to restrict lung cancer development and progression in experimental lung tumorigenesis models (101). Many different approaches are used in various malignancies and other diseases to target IL-6 signaling pathways. For example–small molecules, blocking peptides, and antibodies against IL6, IL-6R, IL6–sIL6R complex, janus kinase (JAK) phosphorylation, and STAT3 activation (102, 103). The upregulation of systemic level of IL6 upon treatment of anti–PD1 antibody nivolumab leads to poor clinical outcome because inhibition of PD1–PDL1 promotes production of IL6 by PD1+ TAMs. Depletion of macrophages *in vivo* model of melanoma reduces the systemic level of IL6 and upregulates anti-tumor Th1 response, suggesting that the narrow therapeutic window of PD1–PDL1 blockade can be overcome by inhibition of IL6 (104).

### TNFα

As the name suggests, TNFα initially found to induce necrosis and cytotoxicity in certain tumors (105). It is also known as a pyrogenic cytokine because of its ability to establish an inflammatory environment in response to pathogens (106). To exert a molecular action on the target cell, TNFα binds to one of the two receptors, TNF receptor superfamily member (TNFR1) (TNFRSF1A, p55TNFR1, p60, or CD120a) and TNFR2 (TNFRSF1B, p75TNFR, p80, or CD120b). According to the molecular context, TNFα exerts an opposite effect on tumor progression. In lung cancer, TNFα found to induce cell proliferation, apoptosis resistance, angiogenesis, invasion, and metastasis in various *in vitro* and *in vivo* lung tumor models (107). On the other hand, doxorubicin treatment-induced TNFα triggers apoptosis of TP53-deficient lung tumor cells via downregulation of cyclin dependent kinase inhibitor 1A (CDKN1A) (108). In the TME, crosstalk of TAMs with tumor cells and other tumor-associated cells via TNFα not only activates survival and proliferation pathways through the transcriptional activation of nuclear factor kappa B subunit 1 (NFKB1), fos proto-oncogene (FOS), and jun proto-oncogene (JUN) but also activates apoptotic pathways via TNFR1. Considering anti-tumor effects of TNFα, number of attempts were made to administer TNFα either systemically or locally in various cancer types. Although administration of TNFα significantly decreased the tumor growth, but many side effects were observed in the studies. In order to augment endogenous TNFα activity, Immunicon Inc. developed a single chain TNFα based affinity column to remove soluble TNF receptors from the blood (109). The pretreatment of low dose of TNFα prior to administration of chemotherapeutic agents such as Cisplatin, Paclitaxel, and Gemcitabine improved the efficacy of the agents in the experimental cancer model (110). On the hand recent studies showed that instead of augmenting effect of TNFα in tumor, TNFα blockade increases effect of immune checkpoint inhibitors (111, 112). Therefore, therapeutic approaches manipulating TNFα in cancer should be interpreted with great caution. The recent studies demonstrated that the higher number of tumor islets with infiltration of TNFα^+^ TAMs (cytotoxic M1 phenotype) confers a survival advantage in non-small-cell lung cancer (NSCLC) and other malignancies (14, 65). In TAMs-tumor cells *in vitro* co-culture model, tumor necrosis factor-related apoptosis-inducing ligand (TRAIL) reprograms TAMs to M1-like phenotype by inducing expression of proinflammatory cytokines like IL1B, IL6, TNFα (113). TAMs-specific TNFα or its receptors induce apoptosis *in vitro* and *in vivo* tumor model by activating CD8^+^ T cells (114). Therefore, current immunotherapeutics need to be directed toward the induction of TNFα^+^ expression in TAMs, thereby reactivating anti-tumor immunity in the TME.

### IL10

IL10 is an anti-inflammatory cytokine mainly produced by activated macrophages, B cells, and T cells (115). IL10 binds to the receptor IL10R. IL10R is a heterotetramer complex consisting of two IL10Rα and two IL10Rβ molecules (116). The main anti-inflammatory functions of IL10 are suppression of classical macrophage activation, suppression of the production of proinflammatory cytokines TNFα, IL1β, IL6, IL8, IL12, and granulocyte-macrophage colony-stimulating factor (GM-CSF) (51, 117), inhibition of antigen presentation by suppressing major histocompatibility complex (MHC) II expression in activated macrophages (118), and inhibition of IFNγ production by Th1 and natural killer (NK) cells (119). Tumor cells often secrete a high amount of IL10, and increased serum concentration of IL10 found to be associated with simultaneous immunostimulation and immunosuppression in different types of cancer (120). The prognostic significance of IL10 in serum or whole tissue homogenate is controversial because a study by De vita et al. suggested that serum IL10 level was a prognostic indicator of advanced NSCLC (66) while studies by Soria et al. demonstrated that patients lacking IL10 expression in early-stage NSCLC have a worse prognosis than those with IL10 expression (67). On the other hand, TAMs-derived IL10 level showed consistent prognostic significance in lung cancer patients (121, 122). TAMs-derived IL10 perform several tasks in lung cancer progression and development (123). Similar to IL6, crosstalk within different cells of the TME via TAMs-derived IL10 leads to the activation of STAT3 (124, 125). Additionally, TAM-derived IL10 promotes the stemness of lung cancer via JAK1/STAT1/NFKB/NOTCH1 signaling pathways *in vivo* tumorigenesis mouse models (126). IL10 also drives *in vivo* lung cancer growth and metastasis by upregulating the CCL2/C-C motif chemokine receptor 2 (CCR2) and C-X-C motif chemokine ligand (CXC3CL1)/C-X3-C motif chemokine receptor 1 (CX3CR1) axis in macrophage-tumor cell crosstalk (20). IL10 signaling pathway is a complex molecular network comprising a minimum of 37 molecules and 76 reactions to support cancer development (127). The blockade of IL10 signaling pathways in human diseases is under critical investigation. Various strategies, such as blocking peptides and monoclonal antibodies against IL10, receptor-blocking strategies for IL10R, and small molecule inhibitors targeting JAK/STAT3 signaling, are under clinical evaluation (128, 129).

## Chemokines

### CCL2

Concerning the potential therapeutic intervention point in various human diseases, CCL2 is one of the most studied chemokines. CCL2 is a potent monocyte chemotactic factor from the C-C chemokine family. It mainly produced by monocyte/macrophages either constitutively or upon induction by other soluble factors and oxidative stress. CCL2 binds to its receptor CCR2 to mediate its effect through an autocrine or paracrine manner (130). Expression of CCL2, CCR2 or in combination with IL6 and IL10 correlates with a worse prognosis in lung cancer patients (61, 78, 79). In various cancers, the crosstalk of TAMs with tumor cells via the CCL2/CCR2 axis play multiple roles in cancer development, such as monocyte/macrophage recruitment at the tumor site (131), tumor progression, EMT, invasion, and metastasis (132). Experimental lung tumor models, *in vitro* TAMs-tumor cells, co-culture models, and human lung cancer biopsies demonstrated that CCL2/CCR2 signaling is one of the central signaling pathways involved in lung cancer growth and metastasis (20, 133, 134). In the *in vivo* metastasis lung tumor model and human lung cancer biopsies, infiltration of TAMs was found to be increased by NFKB1-CCL2 signaling via an elevation in neddylation pathway (135). In the flank and orthotopic lung tumor model, blockade of CCL2 reduces lung tumor growth not only by reprogramming TAMs to M1-like phenotype but also by activating CD8^+^ T cells (136). Deficiency of CCL2 in stromal cells like macrophages reduces infiltration of macrophages, angiogenesis, early tumor necrosis and lung metastasis in the 4T1 breast tumor model (137). Targeting of the CCL2/CCR2 axis in TAMs is an emerging immunotherapeutic tool in various human diseases. Therefore, therapeutic strategies blocking CCL2, CCR2, and CCL2/CCR2 complexes in TAMs are under extensive evaluation (138, 139).

### CX3CL1

To date, CX3CL1 is the only known chemokine from the CX3C family. CXCL1 has received particular attention in chemokine biology because it is found in two forms, either bound to the cell membrane or in a soluble form. Therefore, it acts as both an adhesion molecule and chemoattractant (140). It plays a role in the activation and migration of monocytes, NK cells, T cells, and mast cells at the site of action in various physiological and pathological conditions. It also promotes the binding of leukocytes and the adhesion and activation of target cells. CX3CL1 mediates its cellular effects by interacting with CX3CR1 (141). A recent study by Liu et al. demonstrated that high CX3CL1 mRNA expression served as a positive prognostic indicator in patients with lung adenocarcinoma (80). Another study by Su et al. demonstrated that the CXC3CL1 level showed survival effects in lung adenocarcinoma but not in squamous cell carcinoma (81). The expression of CX3CL1 was found to be increased in lung cancer with higher pathological grades and metastatic lymph nodes (142). CX3CL1-induced M2 macrophage polarization increases invasiveness of human endometrial stromal cells (ESCs) by upregulating expression of MMP9, tissue inhibitor of metalloproteinases (TIMP)-1, -2 and by activating P38MAPK and integrinβ1 signaling (143). In the co-culture of peripheral blood mononuclear cells (PBMCs) and pancreatic cancer cell lines, TRAIL/NFKB1/CX3CL1 dependant bi-directional crosstalk leads to therapy resistance (144). In the mouse mammary tumor model, activation of fibroblast growth factor receptor 1 (FGFR1) leads to migration and recruitment of macrophages via secretion of CX3CL1 (145). In *in vitro, in vivo*, and *ex vivo* models of lung cancer, TAM-tumor cell crosstalk via the CX3CL1/CX3CR1 axis found to be crucial in lung tumor cell growth, and metastasis, suggesting a potential axis for therapeutic intervention in lung cancer (20).

### IL8

IL8/CXCL8 is a proinflammatory chemokine (from the CXC family) mainly secreted by macrophages. It exerts its effect by binding to CXCR1 and CXCR2, which are heterotrimeric G-protein-coupled receptors. These receptors are expressed not only by neutrophils but also by monocytes, endothelial cells, tumor cells, and tumor-associated stromal cells (146). Therefore, IL-8 is responsible for the migration and activation of all these cells (87). IL-8 plays multiple roles in lung cancer development (147–151). IL8 serves as a potential biomarker to predict tumor burden, treatment response, and patient survival in lung cancer (152–154). The expression of IL8 mRNA in the lung TME induced by infiltrating macrophages via the NFKB pathway significantly correlates with increased tumor angiogenesis and shorter median survival of lung cancer (155). IL8 is known to activate major oncogenic signaling pathways through autocrine and paracrine functions in the TME [e.g., PI3K, RAS/mitogen-activated protein kinase (MAPK), and JAK/STAT] (156). Therefore, the precise molecular role of TAM-derived IL8 in TAMs-tumor cell crosstalk requires further investigation. To block IL8 dependent responses, IL8 neutralizing antibodies (ABX-IL8 and HuMax-IL8), small molecule inhibitor of CX3CR1/CX3CR2 (Reparixin, JMS-17-2) are in the different phases of preclinical and clinical development (157, 158).

## Conclusion

The understanding of cytokines and chemokines-mediated interplay between TAMs and tumor cells on a molecular level will allow the development of new immunotherapeutic strategies aimed to unleash the anti-tumor immunity of TAMs in the TME. Given the potential immunomodulatory role of TAM-specific secretory factors in lung cancer development and progression, it is crucial to address the following questions to develop a therapeutic strategy: (i) Are the pro- and anti-inflammation environments triggered by TAM-derived cytokines and chemokines context-dependent? (ii) How does cytokine- and chemokine-based TAM-tumor cell crosstalk influence other immune cells of the TME? (iii) Which are the central signaling pathways regulated by TAM-derived cytokines and chemokines that influence cancer development? A critical evaluation of therapeutic strategies targeting cytokines and chemokines is required to develop potent and efficient immunotherapeutics to restrict cancer development and further validation of these experimental findings in patient samples required for clinical translation.

## Author Contributions

PS, MS, FG, WS, and RS contributed to the conception, writing, and editing for this manuscript.

### Conflict of Interest

The authors declare that the research was conducted in the absence of any commercial or financial relationships that could be construed as a potential conflict of interest.
